# Perspectives on Applications of ^19^F-NMR in Fragment-Based Drug Discovery

**DOI:** 10.3390/molecules29235748

**Published:** 2024-12-05

**Authors:** Qingxin Li, CongBao Kang

**Affiliations:** 1 Guangdong Provincial Engineering Laboratory of Biomass High Value Utilization, Institute of Biological and Medical Engineering, Guangdong Academy of Sciences, Guangzhou 510316, China; 2 Experimental Drug Development Centre (EDDC), Agency for Science, Technology and Research (A*STAR), 10 Biopolis Road, #05-01, Singapore 138670, Singapore

**Keywords:** ^19^F-NMR, fragment screening, drug discovery, fragment-based drug design, in-cell ^19^F-NMR

## Abstract

Fragment-based drug discovery is a powerful approach in drug discovery, applicable to a wide range of targets. This method enables the discovery of potent compounds that can modulate target functions, starting from fragment compounds that bind weakly to the targets. While biochemical, biophysical, and cell-based assays are commonly used to identify fragments, ^19^F-NMR spectroscopy has emerged as a powerful tool for exploring interactions between biomolecules and ligands. Because fluorine atoms are not naturally present in biological systems, ^19^F-NMR serves as a sensitive method for fragment screening against diverse targets. Herein, we reviewed the applications of ^19^F-NMR in fragment screening, highlighting its effectiveness in identifying fragments that bind weakly to various targets such as proteins and RNA. The accumulated evidence suggests that ^19^F-NMR will continue to be a crucial tool in drug discovery.

## 1. Introduction

Drug discovery and development is a time-consuming and high-risk process, beginning with target identification and advancing to clinical drug applications [1,2]. Over 90% drug discovery projects might not be successful due to issues such as efficacy, toxicity, and unsatisfied chemical properties [1,2]. This process usually starts with an early discovery phase to develop a preclinical candidate, followed by a later phase to assess efficacy and other parameters in patients. The development of small molecule drugs carries significant risks, including the low druggability of targets and a low hit rate for novel targets in high throughput screening (HTS) campaigns. However, there are multiple strategies to mitigate these risks in small molecule drug discovery [1]. Selecting a validated target early in a drug discovery project is vital to improve the success rate in clinical trials, as it reduces costs and increases the likelihood of success. Druggability is a concept used to estimate the potential of a small molecule specifically binding to a critical target within key pathways [3]. At the early stages of drug discovery, ligandability is a more suitable term for distinguishing small molecules that specifically bind to a target from those with acceptable pharmacokinetic and pharmacodynamic properties [4]. The ligandability of a target is crucial for predicting the chance of obtaining satisfactory hits in screenings, making it an essential factor in target selection [5].

High throughput screening has been routinely utilized in drug discovery, identifying hits from libraries comprising thousand to millions of molecules [6,7]. While HTS campaigns have led to the development of many drugs, undruggable targets usually result in low hit rates. Enhancing the compound library’s size and diversity can improve hit rates, but this increases operational and hit identification costs. Fragment-based drug discovery (FBDD) offers an appealing alternative to HTS, with benefits like higher hit rates, reduced operational costs, suitability for developing both inhibitors and non-inhibitory molecules that bind to targets [8,9,10], and its synergistic use with other screening methods like HTS and virtual screening [8,11,12] (Table 1). In addition, fragment screening provides useful information to understand the ligandability of a target [9,13].

FBDD differs from HTS campaigns as it begins by identifying compounds that weakly bind to a target [14,15,16] with binding affinity over micromolar [17]. These initial hits are then elaborated into potent compounds for clinical use through various strategies. FBDD offers greater flexibility in the hit-to-lead step since the fragments can be developed into unique structures [8,10,11]. FBDD has been shown to be versatile, applicable to a range of targets including proteins with non-druggable pockets such as KRAS [18,19], enzymes with druggable pockets, protein–protein interactions with shallow binding interfaces, and RNA [8,20]. With seven approved drugs and many lead compounds originating from this approach, FBDD’s role in drug discovery is further validated [21,22]. Moreover, the outcomes of fragment-based screenings can indicate the ligandability of a target, providing an estimate of a drug discovery project’s success rate [3,23]. Evaluating a target’s druggability and potential drug pockets by analyzing the hit rate and binding sites in fragment screening experiments is always prudent [9,13].

In the process of fragment screening, three key elements are considered: the selection of fragment libraries, the strategy for fragment screening, and hit ranking through factors such as ligand efficiency (LE) to evaluate the quality of a fragment [24]. Fragment libraries can be sourced from commercial suppliers, and their small molecular weight, typically less than 300 Da, allows for extensive coverage of chemical space [25,26]. Additionally, novel fragments with specific chemical properties have been synthesized [27]. The ranking of identified hits for further development involves comparing binding affinities, analyzing ligand efficiencies, and determining ligand binding modes [8,11]. Despite the availability of biochemical, biophysical and computational methods for fragment screening, selecting a sensitive method that can detect fragments binding weakly and specifically to a target is crucial [28]. Solution NMR spectroscopy is extensively used in fragment-based screening [9,10,12,29,30], with both ligand-observed and protein-observed NMR experiments being employed [31,32,33,34,35,36,37,38]. ^19^F-NMR is particularly useful for monitoring changes in the ^19^F spectra of fluorinated compounds in the presence and absence of a target, leading to the identification of binding hits [39]. Since fluorine atoms are absent in biological systems and the buffers used for target folding, ^19^F-NMR does not pick up background signals from these sources [35,40].

**Figure 1:**
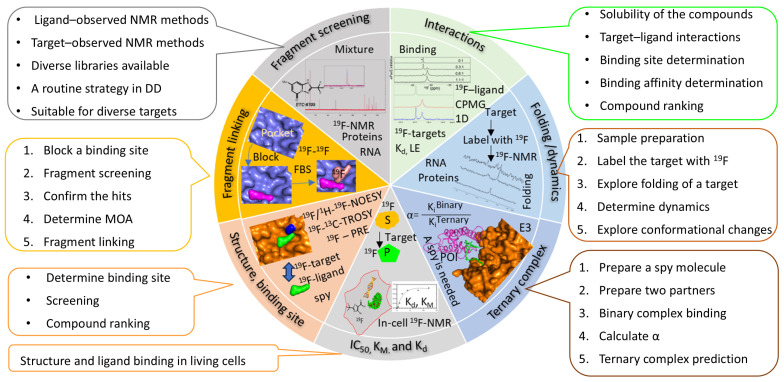
Applications of ^19^F-NMR in FBDD and drug discovery. ^19^F-NMR can be applied to FBDD at different stages. FBS, fragment-based screening; S, substrate; P, product; E3, E3 ligase; POI, protein of interest; CPMG, Carr–Purcell–Meiboom–Gill sequence; TROSY, transverse relaxation-optimized spectroscopy; LE, ligand efficiency; 1D, one dimensional; PRE, paramagnetic relaxation enhancement; NOESY, nuclear Overhauser effect spectroscopy; K_d_, dissociation constant; K_i_, inhibition constant.

The use of ^19^F-NMR in various fields, especially in understanding protein structures and ligand binding, has been extensively reviewed [41,42,43]. This review focuses on the application of ^19^F-NMR in fragment screening. It discusses the strategy and utilization of ^19^F-NMR in fragment-based screening (FBS) against various targets, including proteins and RNA molecules (Figure 1). Accumulated studies demonstrate that ^19^F-NMR is effective for screening fragments against diverse targets, such as those with druggable pockets, membrane proteins reconstituted into membrane systems, and other targets with low ligandability due to the absence of a deep and druggable pocket.

## 2. ^19^F-NMR in Fragment-Based Drug Discovery

The fluorine atom is a 100% abundant isotope and exhibits signal sensitivity comparable to ^1^H NMR [41]. In drug discovery, fluorine atoms are often incorporated into compounds to enhance chemical properties and participate in noncovalent interactions [44,45]. Statistical analysis indicates that many small molecule drugs contain at least one fluorine atom [46,47]. Introducing fluorine atoms into a compound is relatively straightforward during synthesis, making fluorine-containing compound libraries accessible for screening [27,39]. Both commercial and customized libraries with compounds containing one or more fluorine atoms are available for fragment screening [27]. In addition, fluorine atoms can also be incorporated into targets through chemical reactions or by providing amino acid precursors during protein synthesis [21,48]. Several strategies have been developed to produce fluorine-labeled proteins for ^19^F-NMR [49,50,51,52]. With the availability of fluorine-containing compounds and proteins, ligand-, substrate-, and target-based ^19^F-NMR experiments can be applied to fragment screening [33]. It is noted that target-observed NMR experiments are frequently used to explore structures, conformational changes, and dynamics of various proteins and RNA [49,50,51,52,53,54]. In a drug discovery project, both ligand- and target-observed experiments can be used, and these experiments have been described in numerous studies [13,41,55,56].

### 2.1. Ligand-Observed ^19^F-NMR

Compared with proton NMR, ^19^F-NMR is highly responsive to changes in the chemical environment [33]. The chemical shifts of fluorinated compound can span more than 200 ppm [57,58], reducing signal overlap and enabling the measurement of compound mixtures [33,57]. The strategy of using ^19^F-NMR in fragment screening involves selecting fluorinated compound libraries, collecting ^19^F-NMR spectra of compounds in the absence and presence of the target, and ranking the screened compounds for further development [40,59]. While commercial vendors provide fluorinated fragment libraries [33], researchers also create their own libraries tailored to specific features or target types [56].

**Figure 2:**
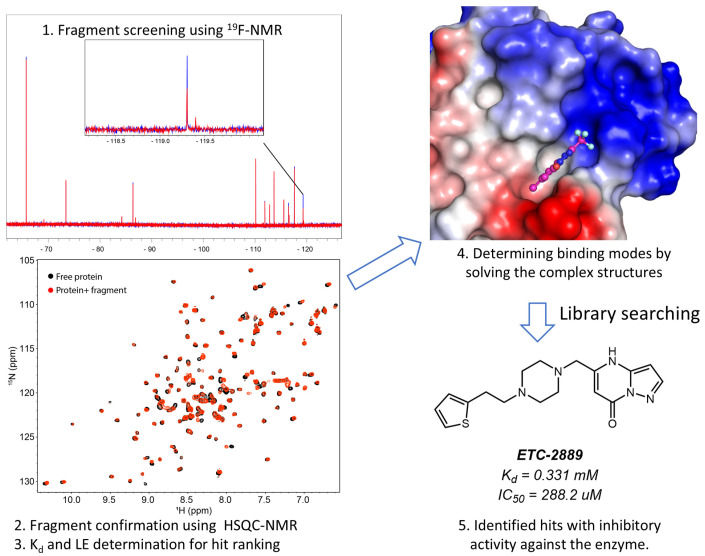
One strategy used for fragment-based drug design. This strategy involves several steps: first, fragment screening using ^19^F-NMR is conducted. Hits are then confirmed through HSQC experiments. Following confirmation, Kd and LE are determined using NMR techniques. The mechanism of action (MOA) is established by solving co-crystal structures. Subsequently, a compound library search is performed to identify compounds with similar fragment structures. Finally, the identified hits are confirmed for further development. The figure was replotted from the reference [65], and permission was obtained.

Ligand-based ^19^F-NMR detects signal changes from a fluorinated compound under different conditions, which is useful for identifying hits and characterizing ligand binding to a target (Figure 1) [41,60,61]. This method can screen hits against a library of fluorinated compounds or identify hits in the presence of a spy molecule—a fluorinated compound with a known binding site [62,63]. The process includes the following steps [17] (Figure 2). First, screening was carried out by utilizing ^19^F-NMR techniques such as standard 1D ^19^F NMR and R_2_-filtered experiments to identify fragments that bind to the target molecule. Second, hit confirmation can be achieved through conducting further biophysical methods, such as ^1^H-^15^N-HSQC experiments and isothermal titration calorimetry (ITC). This step includes evaluating the binding affinities between the fragment hits and the target to rank the identified compounds. Ideally, structural biology techniques are used to understand the binding modes of the hits, which provides insights into fragment growth. Lastly, screened hits are then selected for further development based on criteria such as binding affinity, binding site, ligand efficiency, structural properties, and other characteristics. Due to the high sensitivity of the ^19^F atom to its environment, ^19^F-NMR is highly effective for detecting subtle interactions between fragments and proteins across various concentrations [33,64].

### 2.2. Target-Observed ^19^F-NMR

Fluorine atoms can be incorporated into biological molecules through various strategies for protein-observed ^19^F-NMR experiments [13,55,66]. This incorporation can be achieved by providing precursors during target synthesis or through chemical reactions with purified biological molecules [48,49,67,68,69]. Commercially available building blocks such as aromatic amino acids 3-fluorotyrosine (3FY), 4-fluorophenylalanine (4FF) and 5-flororoindole are commonly used to label proteins for NMR studies and have been used to prepare many proteins for ^19^F-NMR studies [55]. Since aromatic residues like Phe, Tyr and Trp are often present in ligand binding pockets, ligand binding can be measured through monitoring the chemical shift changes of the fluorine atoms attached to these residues [13,55,70]. Resonances in 1D ^19^F-NMR spectra can be well resolved and observed at a low to mid-micromolar concentration. The dissociation constant of a compound with the target can be obtained through a titration experiment. When ligand efficacy needs to be ranked or analyzed, it can be calculated based on the dissociation constant [13]. It is noted that proteins and RNA [71] can be modified with fluorinated compounds for structural and ligand-binding studies. Compared with other protein-observed NMR methods such as ^1^H-^15^N-HSQC, protein-observed ^19^F NMR exhibited some advantages including short data acquisition time, no size limitation for proteins and simple procedure in data acquisition and processing. Regardless of the strategy used to produce fluorinated targets for binding studies, the effect of introducing fluorine atoms on target structure or activity needs to be evaluated before further experiments are conducted.

### 2.3. Fragment Screening Against Proteins

^19^F-NMR has been used for screening fragments that bind to proteins. A recent study demonstrated the application of ^19^F-NMR to identify binders of UBE2T, an E2 enzyme in protein ubiquitin pathway often considered an undruggable target [65]. Several fragments were identified using 1D ^19^F-NMR spectroscopy followed by hit confirmation through protein-observed NMR spectroscopy and X-ray crystallography. Multiple binding pockets in UBE2T were identified, offering valuable insights for structure-based drug design. Additionally, fragment growth was achieved by searching a known compound library containing drug-like molecules, resulting in compounds with inhibitory activity against UBE2T.

Another study utilized ^19^F-NMR to identify fragments that could disrupt the BRCA2–RAD51 interaction, a crucial target in cancer drug discovery. Fragment screening to identify RAD51 binders was conducted using a ^19^F T_2_ filtered NMR experiment. Screening was performed with a mixture of 20–25 fragments by comparing the spectra in the absence and presence of RAD51. To identify fragments that could bind to the protein–protein binding interface, a BRC4 peptide was used for selection. Further chemical modifications led to the development of a potent compound **46** that exhibited activities in pancreatic cancer cell lines through affecting protein–protein interactions [72]. This developed compound can serve as a chemical probe to further evaluate the function of RAD51/PARP1-2 and as a starting point for developing inhibitors. ^19^F-NMR has been applied to screen fragments binding to diverse proteins (Table 2). The identified fragment from this approach can also be used as a spy molecule for screening of libraries with fragments or drug-like molecules [73]. A recent study utilized a fluorinated compound designed based on the crystal structure as a probe to investigate drug binding to a G protein-coupled receptor [74], further demonstrating the effectiveness of this approach.

### 2.4. Fragment Screening Against RNA

RNA has emerged as a novel class of drug targets due to its significant role in various diseases [84,85]. Targeting viral RNA is vital for combating infectious diseases, especially with the health risks posed by RNA viruses [86]. Although traditionally seen as undruggable due to their nature, advancements have led to small molecules capable of modulating RNA functions [87]. This has spurred drug discovery initiatives aimed at different RNA types [88,89]. FBDD plays important roles in developing binders of RNA to modulate their functions [90]. Assays to identify small molecules that bind to RNA have been developed [91,92], with NMR spectroscopy becoming an important tool for analyzing RNA-ligand interactions [71,93,94]. Similarly to proteins, fluorine-labeled RNA can track interactions with ligands via ^19^F-NMR [95]. Ligand-observed ^19^F-NMR has also been used to detect fragment binding to RNA [96]. A study utilized ^19^F-NMR to screen a library of 102 fragments against 14 RNAs of varying sizes and sequences, including elements like small step loops, riboswitches, and tRNAs [61]. This research provides a thorough review of fragment screening for diverse targets using ^19^F-NMR. It is crucial to acknowledge that RNA structure and dynamics can change under different conditions, making it imperative to verify RNA folding before starting screening processes.

### 2.5. ^19^F-NMR for Fragment Linking

According to one of the principles of FBDD, linking two fragments with binding affinities in the millimolar range can result in a more potent compound with micromolar affinity, making fragment linking a promising strategy for fragment growth [10]. Several potent compounds have been developed using this approach [15,26,78]. However, identifying two fragments in close proximity within a binding pocket is challenging. To address this, a “second-site” approach using ^19^F-NMR was developed and successfully applied to create BACE-1 inhibitors [97]. In this study, one part of the BACE-1 pocket was initially blocked with a well-studied compound. ^19^F-NMR screening was then performed to identify fragments binding to the “second site,” another portion of the pocket. Molecular docking and NMR experiments guided fragment growth, resulting in compounds with improved potency and selectivity. This strategy is particularly useful in target-based drug design, especially for targets with known ligands.

### 2.6. Substrate-Based ^19^F-NMR

A novel method known as n-FABS (n-fluorine atoms for biochemical screening) has been developed for monitoring substrate changes during enzyme catalysis using ^19^F-NMR [98,99]. This technique involves labeling the substrate with fluorine atoms, such as a CF_3_ moiety, for ^19^F-NMR measurement. By tracking changes in the ^19^F-NMR spectrum of the ^19^F-labeled substrate during the enzymatic reaction, ^19^F-NMR spectroscopy can determine enzyme parameters like K_M_ and screen compound libraries for inhibitors [100]. The method offers several advantages, including high sensitivity from CF_3_ labeling, no interference from buffers and enzymes due to the absence of natural fluorine atoms, flexibility in screening inhibitors with varying activities by adjusting substrate concentrations, suitability for high-throughput screening with compound mixtures, and applicability for screening a wide range of compounds, including fragments and drug-like molecules. This method can also generate reliable IC_50_ values [101], critical for determining the structure–activity relationship (SAR) of a series of compounds. It can be applied to diverse systems, from purified enzymes including membrane proteins [81] to cell lysates and intact cells [100]. In addition to labeling substrates with a CF_3_ group, a fluorinated ATP analog, 2-fluoro-ATP, has been used in ^19^F-NMR experiments. This analog can be used to screen compounds that inhibit or activate reactions [102].

## 3. Determining Binding Affinity by ^19^F-NMR

Ranking the identified hits in fragment-based drug design is crucial for advancing from hits to leads. While comparing IC_50_ values of hits using biochemical assays is important, many fragment hits may not exhibit measurable values due to their low binding affinity with the target. Several biophysical methods, such as ITC and SPR, are available for measuring binding affinities. However, NMR spectroscopy is a valuable tool for exploring ligands binding weakly to targets [103,104].

**Figure 3:**
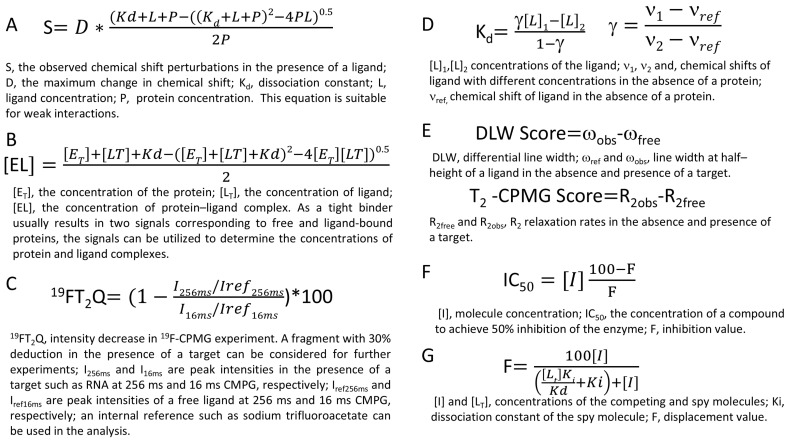
Measure-binding affinity using ^19^F-NMR. (**A**) One equation to determine binding affinity. Protein-observed NMR was used to determine binding affinity, which is suitable for weak binding (Kd > 1 µM) [13,107]. (**B**) One equation to determine ligand binding. Protein-observed ^19^F-NMR is used, which is suitable for strong ligand (Kd < 1 µM) [103]. (**C**) One equation used to identify fragment hits. The effect of a ligand on line broadening is commonly used in fragment screening [76]. (**D**) Ligand-observed ^19^F-NMR to estimate ligand binding affinities. The binding affinities can be estimated by monitoring CSPs or line broadenings at two different ligand concentrations [108]. (**E**) ^19^F differential line width (DLW) score and T_2_-CMPG score are used to evaluate ligand-binding affinities. These scores provide more reliable evidence to rank the identified fragments, especially in the hit-to-lead step and understanding structure–activity relationship [83]. (**F**) A method to calculate IC_50_ of an enzyme using ^19^F-NMR. A titration experiment is needed to obtain the value [100]. (**G**) Determine binding affinity with a competition experiment. A spy molecule is needed for measuring binding affinity of a compound [109,110]. Please refer to the references for experimental details.

Affinity of a fragment can be evaluated through direct and indirect strategies [103]. In direct measurements, both protein- and ligand-observed ^19^F-NMR approaches can determine binding affinities. In protein-observed experiments, titrating the ligand into the ^19^F-labeled target can be utilized to determine binding affinity (Figure 3A) [103]. For ligand binding resulting in line broadening, changes in lineshape can be utilized to determine binding affinity through lineshape analysis (Figure 3B,C) [105]. For ligands inducing chemical shift perturbations (CSPs), binding affinity can be determined by analyzing CSPs caused by different fragment concentrations [13]. Ligand-observed ^19^F-NMR methods are available to rank hits. Chemical shift-anisotropy-based affinity ranking (CSAR) is a reliable method to rank hits derived from ^19^F-NMR [106]. Ligand-observed ^19^F-NMR can also determine dissociation constants by measuring spectra at two ligand concentrations, [72] among other reviewed methods (Figure 3D,E) [103].

Binding affinities can also be estimated using indirect methods, in which a spy or a reference compound is needed [103]. ^19^F-NMR can rank identified fragments by measuring their binding affinities or determining their IC_50_s when a ^19^F-labled spy molecule is available (Figure 3F,G). The binding affinity between the target and fragments can be assessed using ^19^F-NMR. When the binding affinity or IC_50_ of a fragment is available, ligand efficiency (LE) can be determined and used as one criterion for hit selection. A suitable strategy to rank fragments should be selected, as drug discovery projects usually have defined timelines. Besides ranking fragments using calculated LE values, other factors such as fragment binding sites and structures are also considered in hit selection.

## 4. Perspectives

FBDD is a powerful way to develop novel compounds targeting different proteins or RNA [25]. Although extensive chemical modifications are necessary to enhance the potency of the initial fragment, FBDD represents a unique strategy for developing strong binders for a target and can be used in combination with other approaches to enhance the efficiency of the drug discovery process. ^19^F-NMR will be playing important roles in FBDD by participating in screening, ranking hit binding affinities, mapping hit binding sites, evaluating binding affinities and providing novel hits for fragment growth. Despite its advantages, ^19^F-NMR faces certain challenges. The impact of fluorine atoms in compounds on their binding to the target must be assessed by synthesizing analogs or determining the structures of complexes. If binding is influenced by the introduced fluorine atom, a thorough evaluation of the compounds becomes necessary. Additionally, functional analysis of a fluorinated target is essential prior to experimentation, as the modification may affect the target’s folding and functionality. The following elements are important for ^19^F-NMR in FBDD [111,112].

### 4.1. Fragment Libraries

The selection of libraries for screening is crucial in fragment-based drug discovery [26,57]. When selecting a library for screening, several factors should be considered, including the number of fluorine atoms in the fragments, chemical diversity, fragment complexity, unique structural features, solubility, potential for aggregation, fluorine positioning, and the presence of specific functional groups. Numerous commercially available fluorinated fragment libraries offer a wide range of chemical structures for developing potent compounds against diverse targets. Developing libraries diverse in shape and containing natural-product-like compounds with desirable physicochemical properties can expand the chemical space available for drug discovery, particularly for targets with challenging pockets that are traditionally difficult to drug [27,57].

### 4.2. ^19^F-NMR in Fragment Screening

Fragment screening using ^19^F-NMR is an efficient and cost-effective approach applicable to a variety of targets. Its rapid turnaround time, typically completed within weeks, makes it an attractive method for target-based drug discovery projects. Even when a high-throughput screening campaign is planned in a drug discovery pipeline, ^19^F-NMR fragment screening can be beneficial. It provides insights into the target’s druggability and identifies a spy molecule for determining the binding site of hits from other screening campaigns. The hit rate obtained from fragment screening can inform and guide subsequent HTS activities. Compared with other methods such as thermal shift assay and surface plasmon resonance, ^19^F-NMR is more suitable for fragment screening due to its capability to detect weak binders and utilization of fragment mixtures in the screening. This approach offers a highly promising strategy for screening hits that bind to membrane proteins, particularly in comparison to other biophysical methods. Its advantage lies in the fact that the membrane-mimicking systems employed during protein purification do not influence the experimental outcomes. This method is well suited for screening fragments with weak binding affinities (K_d_ > µM) [17]. However, it is less effective for identifying fragments that bind strongly to the target. For large protein complexes or highly dynamic systems, more careful design is needed due to the complexity of data interpretation.

### 4.3. Determining Ligand-Binding Sites

Determining ligand-binding sites using ^19^F-NMR can be achieved through various strategies. One approach involves labeling the target protein or RNA with fluorine atoms and using protein-observed ^19^F-NMR to locate the binding site. Alternatively, a fragment or compound with a known binding site can be fluorinated to create a reference molecule that identifies other ligand binding sites through competitive experiments. Additionally, in ligand-observed fragment screening using ^19^F-NMR, the binding site of a fragment hit can be determined by adding a substrate or known binder of the target. Although this method differs from obtaining high-resolution structures of target–ligand complexes, it remains valuable for targets where co-crystallization is challenging. ^19^F-NMR can also be used to understand the structure–activity relationship of compounds when a reference compound is available [113]. When a binding pocket is blocked by a compound, ^19^F-NMR can be utilized to identify a secondary binding site [82]. This method can also be used based on the known structural information such as binding information derived from computational modeling. Computation prediction and ^19^F-NMR screening was used to identify an allosteric pocket in lectin [114,115]. Overall, determining ligand-binding sites using ^19^F-NMR can be achieved through direct competitive experiments with a known compound, mapping sites using a fluorine-labelled protein, identifying hotspots through blocking a known pocket and identifying allosteric pockets through combination with other methods.

### 4.4. ^19^F-NMR in Determining Structures of Target–Fragment Complexes

^19^F-NMR plays a significant role in the structural biology of targets, small molecules [116], and target–ligand complexes. Although the restraints derived from ^19^F-NMR experiments are limited, they have been efficiently applied to study the folding of macromolecules under various conditions. Restraints derived from ^19^F-NMR experiments, such as ^19^F-NMR paramagnetic relaxation enhancement experiments (PREs) [117] and ^1^H/^19^F-^19^F NOE experiments [41], provide valuable structural information for understanding the structures of targets and their complexes with ligands. This information can be further applied to docking methods to provide reliable information to understand the binding modes of small molecules.

### 4.5. Fragment Screening Using ^19^F-NMR in Cells

Monitoring of target–ligand interactions in living cells can be achieved through in-cell NMR experiments [118,119,120]. Despite challenges like low signal sensitivity and the crowded cellular environment, various NMR experiments have been successfully conducted in living cells, yielding spectra with satisfactory signal-to-noise ratios [110,121]. In-cell ^19^F-NMR has been specifically used to monitor protein–ligand interactions in real-time. Furthermore, when a reference molecule is available, ^19^F-NMR can facilitate compound screening to identify drug-like molecules through competitive experiments [110,122]. In-cell ^19^F-NMR provides a strategy to explore target–ligand interaction in the native environment [123], which could monitor interactions in real time. In addition, drug uptake and distribution can also be investigated using in-cell ^19^F-NMR. This method also makes it possible to investigate protein/RNA folding and dynamics in living cells. Its ability to monitor drug and target interactions in living cells could be useful in exploring target engagement and bridging the gap between in vitro studies and in vivo applications.

### 4.6. Competitive Binding with ^19^F-NMR

Screening a library of fragments or drug-like small molecules and evaluating the potency of compounds can be accomplished through ^19^F-NMR competition experiments [124]. This strategy requires a reference or a spy molecule that is fluorinated. A fragment hit identified using ^19^F-NMR can serve as a spy molecule to perform further screening experiments or evaluate the potency of developed compounds during the hit-to-lead discovery stage. However, not all fragments are suitable as spy molecules. Factors such as binding affinity, mechanism of action, and sensitivity must be considered. One study outlined the strategy for selecting an appropriate spy molecule for ^19^F-NMR studies [62].

### 4.7. Fragment Linking Supported by ^19^F-NMR

Fragment growth can be achieved through several strategies, such as fragment linking, fragment merging, or a combination with computational-aided drug design strategies [14,125,126,127]. Fragment linking is an efficient way to improve the potency and specificity of the fragment hits [128]. However, this strategy is hindered by the challenges in identifying fragments that are in close proximity. ^19^F-NMR has been shown to play a role in fragment linking by serving as an important way to screen fragments binding to a known site whose nearby pocket is blocked by a known fragment. Additionally, ^19^F-^19^F-interligand NOE (ILOE) experiments can confirm the close contact of the identified fragments in one pocket. If the protein is fluorine-labeled, ^19^F-^19^F-NOE can also be used to confirm the fragment-binding mode.

### 4.8. Develop a Reference/Spy Molecule for Assays Using ^19^F-NMR

Small molecules, such as PROTACs and molecule glue, can induce ternary complex formation, offering a new approach to drug discovery [129]. It is crucial to monitor this process to understand the mechanisms of action of developed molecules in a drug discovery project [130]. Various methods have been developed for monitoring ternary complex formation [131]. While not all targets can be crystallized due to challenges such as protein dynamics, solving the structure of the ternary complexes is a direct strategy to assess the capability of the developed compounds. NMR spectroscopy is a powerful tool for monitoring protein complex formation in solution [132]. For instance, ^19^F-NMR competition binding experiments were conducted to evaluate the cooperativity of PROTACs, demonstrating the capability of ^19^F-NMR to estimate both positive and negative cooperativity of a PROTAC when a spy molecule was present [133]. Therefore, it is useful to identify a spy molecule, such as through fragment screening using ^19^F-NMR. The identified fragment hits can serve as reference compounds for monitoring the activity of a PROTAC or similar molecule, which is important for developing small molecules to induce the formation of ternary complexes.

### 4.9. Overcoming Challenges in ^19^F-NMR

To overcome challenges encountered in ^19^F-NMR to expand its application across a broader range of applications in drug discovery, the following elements can be considered. First, enhancement in NMR data collection through introducing new pulse programs and more sensitive probes. Second, the development of more sensitive compounds and probes for binding assays. Third, integration with other biophysical methods such as X-ray crystallography, SPR and even computational methods. Lastly, applications of automation and throughput will further enhance the efficiency in screening campaigns.

## 5. Summary

^19^F-NMR appears to be a useful tool in FBDD due to its high sensitivity to changes in the chemical environment and wide chemical shift range, which reduces signal overlap and enables the analysis of complex mixtures. The method’s flexibility in screening diverse compound libraries and its application to challenging targets make it an indispensable technique in modern drug discovery (Figure 1). With the development of in-cell NMR experiments, ^19^F-NMR can be readily applied to probe target–ligand interactions in living cells.

## Figures and Tables

**Table 1:** Some differences between HTS and fragment-based screening (FBS).

Items	HTS	FBS
Compounds in the library	Molecules following rule of five	Fragments following rule of three
Molecular weight	~500 Da	<300 Da
Size of a library	>10,000	Smaller sizes between 100 and 10,000
Compound storage and processing	Automation and a special place	Manual and normal fridges
Assays used in screening	Activity/phenotype-based assays	Biophysical assays
Assay development	Needed for an individual target	General methods can be applied
Screening strategy	Usually, one compound per well	Mixture of fragments
Target identification	No need for phenotypic screening	Required in screening
Hit rate	Low	High
Hit potency to a target	High	Low
Hit binding affinity to a target	High (K_d_ in µM-nM)	Low (in mM-µM)
Structure-activity relationship (SAR)	SAR needs to be explored	SAR known during the hit-to-lead step

**Table 2:** Some examples showing the applications of ^19^F-NMR in fragment screening.

Target	Fragments in Screening	Experiment	Outcomes	Ref.
UBE2T	Mixture of 10 fragments.	1D ^19^F-NMR	Hit rate: 2.5%	[65]
BRCA2-RAD51	Mixture of 20–25 fragments	^19^F-CPMG	Two hits were identified, and one potent compound was developed.	[72]
FKBP12 GSK3 JAK3 VSOP	Mixture of compounds, competitive assay	^19^F-filtered NMR and ^19^F-DOSY	Hit rate: 1.4% Hit rate: 7.2% Hit rate: 4.1% Hit rate: 6.3%	[17]
14 RNAs	Mixture of 20 or 21 compounds	^19^F-CPMG	Fragments binding specifically to a target can be identified.	[61]
TERRA	Mixture of eight compounds	^19^F-CPMG and 1D	Hit rate: 5.6%	[75]
SARS-CoV-2 RNA	Different RNA constructs and concentration of the fragment used	^19^F-CPMG and 1D NMR	Hit rate: 20.4%	[76]
Riboswitch and protein tyrosine kinase	Mixture of 20 fragment	1D ^19^F-NMR and CPMG	Detailed procedures are provided in a video format.	[77]
Brd4 and BrdTt, and the KIX domain.	^19^F-labeled protein mixed with fragment mixtures	Protein observed ^19^F-NMR	Steps required for FBS using protein-observed NMR were described.	[13]
p70S6K1 and p38g, BACE-1, and DC-SIGN,	Mixture of 20–22 Fsp3-rich fragments	^19^F-CPMG	A 3F library was developed, and hit rate ranged from 3 to 11%.	[27]
BACE-1	20 µM fragment	1D ^19^F-NMR	A screening was performed in the presence of a blocking compound, resulting in identification of fragments binding to a different pocket.	[78]
CBP/p300 KIX domain	Mixture of six compounds	Protein-observed NMR	Hit rate: 0.4%	[79]
Brd4	Mixture of five compounds	Protein-observed NMR	Comparation with ^1^H-CMPG in screening was made.	[80]
Membrane enzyme FAAH	Mixture of five compounds	n-FABS	Hit rate:16.5% IC_50_ of hits were identified.	[81]
4′-phosphopantetheine adenylyltransferase (PPAT)	Reference compounds were used to identify hot spots of a target.	^19^F-CPMG	^19^F reporters binding to hot spots were generated and used for screening.	[82]
BPTF and PfGCN5	Mixture of four–five fragments	Protein-observed NMR	Hit rate: 9.2% and 9.8%	[60]
Bacterial COaD	Mixture of 35 fragments	BURBOP experiment	A new pulse program was developed to increase number of fragment screening.	[58]
HRAS	Mixture of 10–15 fragments	1D ^19^F-NMR	Hit rate: 3%	[83]

## Data Availability

Not applicable.
